# Simulations of Structural Assembly in Charged Dendrimers with Surfactants

**DOI:** 10.1021/acs.jpcb.5c00724

**Published:** 2025-04-24

**Authors:** Jarosław S. Kłos, Sebastian Wołoszczuk, Jarosław Paturej

**Affiliations:** † Faculty of Physics, A. Mickiewicz University, Uniwersytetu Poznańskiego 2, 61-614 Poznań, Poland; ‡ Leibniz-Institut Für Polymerforschung Dresden e.V., 01069 Dresden, Germany; § Institute of Physics, University of Silesia in Katowice, 75 Pułku Piechoty 1, 41-500 Chorzów, Poland

## Abstract

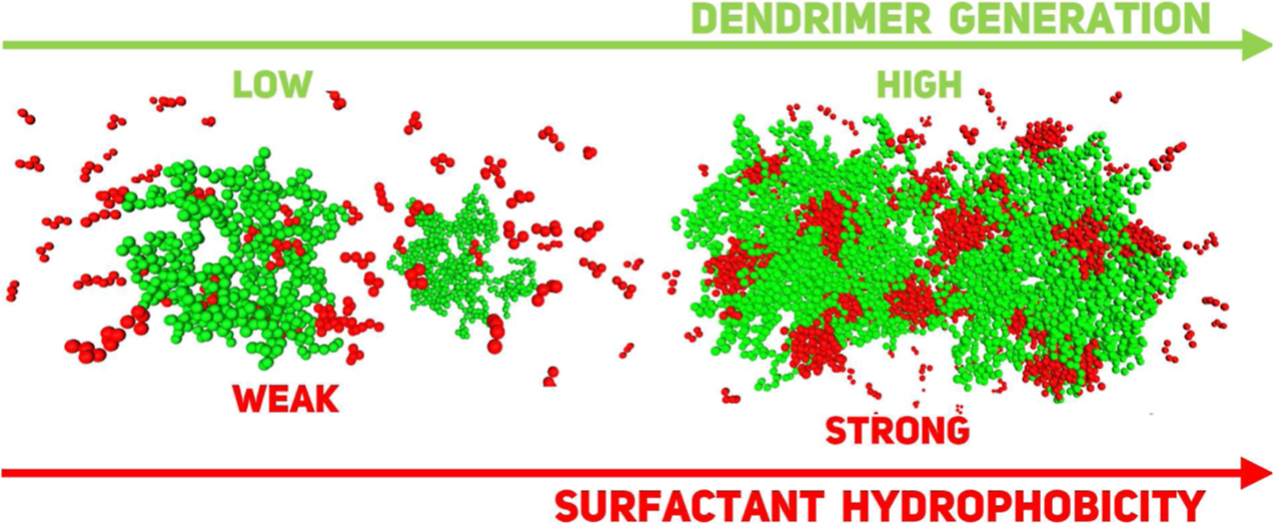

Using Langevin dynamics simulations, we studied the formation of complexes among two cationic dendrimers, anionic surfactants, and counterions. The system includes free dendrimers, surfactant micelles, and mixed clusters, where micelles bind to dendrimers. These micelles are categorized into bridge micelles, which connect two dendrimers, and corona micelles, which are bound to individual dendrimers. We observe that more hydrophobic surfactants exhibit a stronger tendency to assemble with each other and dendrimers, reducing the number of free micelles, which predominantly exist as unimers while enlarging both bridge and corona micelles. Most counterions remain uncondensed and diffuse in solution. On this basis, we conclude that aggregate formation resembles an ion exchange process, where large micelles act as multivalent ions, replacing counterions in their assembly with dendrimers. Additionally, we introduce aggregate indexing, represented by pairs of integers, specifying the number of dendrimers and surfactants in aggregates. A histogram of aggregate counts for each index is used to analyze the aggregates in detail. We find that various mixed clusters occur with specific probabilities, including statistically favored clusters defined by their most probable composition. We also observe that, for weakly hydrophobic surfactants, one-dendrimer clusters dominate, while strongly hydrophobic surfactants and high-generation dendrimers form two-dendrimer clusters. Furthermore, the aggregates are characterized by the mean number and mass of the bridge and corona micelles they contain as well as their mean effective charge. The favored mixed clusters typically contain a small number of micelles per dendrimer, with the micelle mass increasing as the surfactant hydrophobicity increases. The effective charge of the aggregates primarily depends on the combined charge of dendrimers and surfactants with minor contributions from counterions. For high-generation dendrimers, the charge remains positive, while for lower-generation dendrimers, it shifts from positive to negative at a specific number of complexed surfactants. In particular, charge inversion is also observed in the favored mixed clusters.

## 1 Introduction

The interactions between dendritic polyelectrolytes and ionic surfactants give rise to diverse aggregate structures, as revealed by various experimental techniques.^1^ Nuclear magnetic resonance (NMR) studies demonstrated the formation of surfactant inclusions in the intermolecular interactions between dendrimers and surfactants.^2^ NMR and NOE measurements also showed the formation of micelle-like aggregates on dendrimer surfaces, with the structure and dynamics of these complexes influenced by surfactant concentration and dendrimer generation.^3^ At low pH, electrostatic interactions drive the formation of stable dendrimer–surfactant complexes, while at higher pH, weaker binding results in the formation of smaller or resoluble aggregates.^4^ Small-angle neutron scattering (SANS) showed that lower-generation dendrimers promote spherical micelle formation, whereas higher generations lead to larger, less densely packed aggregates due to steric constraints.^5,6^ Additionally, dynamic light scattering (DLS) and isothermal titration calorimetry (ITC) confirmed that dendrimer–surfactant binding occurs at surfactant concentrations below the critical micelle concentration, emphasizing the role of cooperative interactions.^7^ Microscopic and scattering techniques, including transmission electron microscopy (TEM),^8^ cryogenic electron microscopy (cryo-EM),^9^ and small-angle X-ray scattering (SAXS),^10−12^ highlighted structural transitions from lamellar phases to dynamic nanotubes and ribbon phases. These transitions were sensitive to the surfactant concentration, dendrimer generation, and binding ratios. For instance, lamellar structures were observed in complexes with low-generation dendrimers, while higher generations produced compact surfactant–dendrimer assemblies. Further investigations demonstrated practical applications of polymer–surfactant assemblies in biology and medicine. Recent studies showed that surfactant–polymer complexation at interfaces can significantly influence crystallization processes, affecting molecular packing and structural order in drug formulations.^13^ Green dendrimer–surfactant complexes revealed enhanced drug loading and gene transfection capabilities,^14^ while specific combinations, such as PAMAM dendrimers with anionic surfactants, mimicked nucleosome-like structures.^15^ The ability to modulate these supramolecular architectures by adjusting the pH, dendrimer generation, or surfactant concentration underscores their potential as versatile platforms for targeted material design.

In our previous works,^16,17^ we used molecular dynamics simulations and a bead–spring coarse-grained model to investigate complexes of single cationic dendrimers, anionic surfactants, and counterions. We found that the surfactant concentration and hydrophobicity govern three distinct structural regimes. Weakly hydrophobic surfactants bind to dendrimers noncooperatively as unimers and exist in bulk solution in the same form. Moderately hydrophobic surfactants exhibit cooperative binding, forming micelle-like aggregates on dendrimers, with unimers persisting in the solution. Highly hydrophobic surfactants form multichain aggregates within dendrimers and in the bulk at higher concentrations. The formation of these large aggregates significantly alters the system, driving a shift in the overall net charge of the aggregates from positive to negative.

Building on our understanding of the structure of complexes formed by single dendritic polyelectrolytes and ionic surfactants and incorporating the experimental findings discussed earlier, this paper extends the simulation study to systems with two terminally charged dendrimers. Specifically, we investigate pairs of dendrimers from three generations, immersed in ionic surfactant solutions with varying degrees of hydrophobicity, ranging from weak to strong. These studies investigate how molecular weight, dendrimer architecture, charge, and intersurfactant attractions affect complex formation, revealing clustering and cooperativity in multidendrimer assemblies. This approach enables us to explore the formation of both single-dendrimer and two-dendrimer complexes, along with the collective phenomena that emerge when dendrimers interact with oppositely charged surfactants. By studying multidendrimer systems in surfactant solutions, we aim to gain deeper insights into aggregation, cluster formation, and the role of intermolecular interactions in defining the properties of these systems.

This paper is organized as follows. In Section 2, we outline the model and the simulation method. Our results are presented and discussed in Section 3. We draw conclusions and remarks in Section 4.

## 2 Simulation Model

We perform implicit-solvent coarse-grained Langevin dynamics simulations of three systems, each containing *n*_*d*_ = 2 terminally charged dendrimers (*G*3*S*4, *G*5*S*4, and *G*7*S*4) and *n*_*s*_ = 600 surfactant chains. In the above acronyms, *G* stands for dendrimer generation and *S* for the spacer length, expressed in the number of bonds joining adjacent beads along a spacer chain. The tree-like structure of the dendrimers includes a core made up of two bonded monomers and trifunctional branching groups. The overall number of the monomers, *N*_*d*_, and the terminal groups, *N*_t_, are given by^18,19^
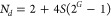
1and
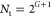
2

**Figure 1:**
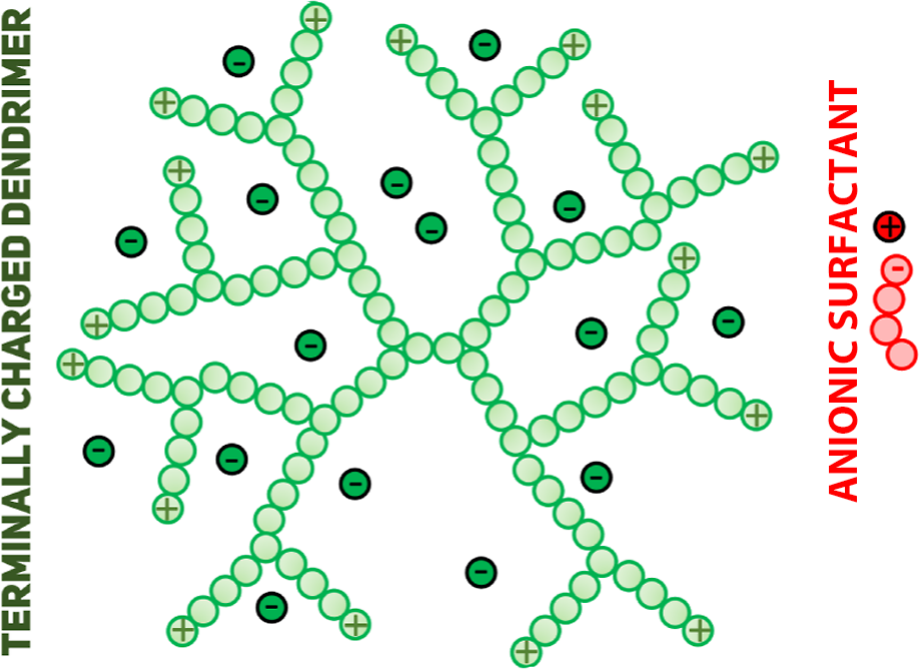
Schematic representation of the coarse-grained model of a cationic *G*3*S*4 dendrimer (shown in green), an anionic surfactant (shown in red), and counterions.

We assign monovalent positive charges to the terminal groups of the dendrimers to match the charge distribution found in poly(amidoamine) (PAMAM) cationic dendrimers at neutral pH.^20^ The anionic surfactant molecules are modeled as chains comprising a negatively charged monovalent head bead and three hydrophobic tail monomers. To ensure overall system neutrality, the model also includes the appropriate number of mobile anions and cations (termed dendrimer and surfactant counterions, respectively). The schematic representation of a model comprising a terminally charged *G*3*S*4 dendrimer, a head-charged surfactant, and the corresponding counterions is shown in Figure 1. In the simulations, we incorporate three types of interactions between a pair of particles separated by a distance *r*. The Lennard-Jones (LJ) 12–6 truncated and shifted potential
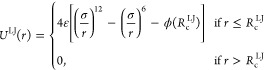
3where , ε is the interaction strength, σ is the diameter of the monomers, and *R*_c_^LJ^ is the cutoff radius. The bonds between adjacent polymer beads are modeled using the Finite Extensible Nonlinear Elastic (FENE) potential
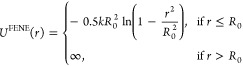
4where *k* is the spring constant and *R*_0_ is the maximum extension of the bonds. Electrostatic interactions are introduced using Coulomb pairwise potential
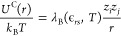
5where *k*_B_ denotes the Boltzmann constant, *T* the absolute temperature, λ_B_ the Bjerrum length of the solvent, and *z*_*i*_ and *z*_*j*_ (±1) the charge valence of the *i*th and *j*th particles, respectively. The Bjerrum length is defined as λ_B_(*ϵ*_*rs*_,*T*) = *e*^2^/(4πϵ_0_*ϵ*_*rs*_*k*_B_*T*), where ϵ_0_ is the electric permittivity of the vacuum, ϵ_*rs*_ is the relative permittivity of the solvent, and *e* is the elementary charge. Our simulations involve dendrimers and surfactant chains immersed in water (ϵ_*rs*_ = ϵ_*rw*_ ≈ 81) at room temperature (*T*_r_ ≈ 298 K). This results in λ_B_(ϵ_*rw*_,*T*_r_) ≈ 7Å.

The LAMMPS molecular dynamics package^21^ was used to perform Langevin dynamics simulations in reduced Lennard-Jones units, with the electric permittivity of the solvent set to ϵ_*rs*_ = ϵ_*rw*_ = 81. To set the unitless simulation parameters (denoted with an asterisk), ε_*u*_ = 1*k*_*B*_*T*_*r*_ and σ_*u*_ = λ_B_(*ϵ*_*rw*_,*T*_r_) were used as the real units of energy and length, respectively. For the FENE potential, the parameters were k* = *k*σ_*u*_^2^/ε_*u*_ = 30 and *R*_0_^*^ = *R*_0_/σ_*u*_ = 1.5.^22^ According to the formula for the reduced elementary charge, , and for the reduced temperature, T* = *k*_B_*T*/ε_*u*_, we set these parameters to e* = 9 and T* = 1. To model attractions between the surfactant tail beads, the cutoff radius, *R*_c_^*LJ^ = *R*_c_^LJ^/σ_*u*_ = 2.5, was introduced in the LJ potential described in eq 3. In our simulations, we varied the strength of attraction between the tail beads by using three values for ε* = ε/ε_*u*_: 1.25, 1.5, and 1.6. These values span the range from weak to strong hydrophobic attractions. For interactions between all other particles, the strength was set to ε* = 1 and the cutoff to *R*_c_^*LJ^ = 2^1/6^, resulting in purely repulsive LJ interactions that mimic good solvent conditions. The diameters of all particles were set to σ* = σ/σ_*u*_ = 1. Coulomb interactions were calculated using the Particle–Particle–Particle Mesh (PPPM) method, with an error tolerance for forces of 10^–4^ and a real-space cutoff radius of *R*_c_^C*^ = 10.^21^ The damping parameter in the Langevin equation of motion was set to γ* = γ/*t*_*u*_ = 1, where  is the LJ time unit and *m*_*u*_ is the assumed real mass unit. For example, setting *m*_*u*_ ≈ 30 g/mol results in *t*_*u*_ ≈ 2.4 ps. The simulations were performed with a time step of Δt* = Δ*t*/*t*_*u*_ = 0.005 and m* = *m*/*m*_*u*_ = 1 for all particle types. In this paper, we present results for systems with a surfactant concentration of , fixed through the periodic boundary conditions of a cubic simulation box with a length of L* = 70. At this concentration, our model ionic surfactant readily forms micelles over a wide range of reduced hydrophobicity ε*. Typically, about 10^6^ integration steps were used for equilibration, followed by 10^7^ integration steps for the production runs. The simulation snapshots were rendered using the Visual Molecular Dynamics.^23^

## 3 Results

**Figure 2:**
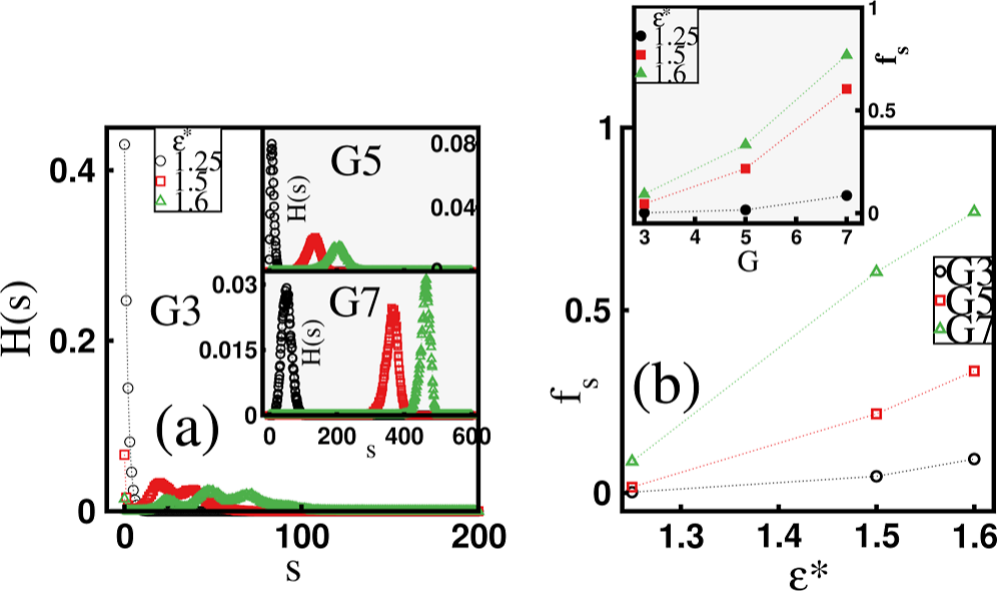
(a) Histogram, *H*(*s*), of the number of absorbed surfactants by dendrimers, *s*, for varying surfactant hydrophobicity, ε*, and fixed dendrimer generation, *G*. (b) The mean fraction of absorbed surfactants, *f*_*s*_, as a function of ε* for a given *G*. The inset in (b) shows *f*_*s*_ as a function of *G* at a fixed value of ε*.

Our simulations reveal that anionic surfactants and cationic dendrimers form complexes driven by hydrophobic attractions among surfactant molecules and electrostatic attractions between the dendrimer terminal groups and surfactant head groups. To quantify the degree of surfactant association with dendrimers, in Figure 2a, we present the histogram, *H*(*s*), of the number of surfactant chains, *s*, absorbed by dendrimers for fixed hydrophobicity, ε*, and dendrimer generation, *G*. Note that in this plot we do not distinguish between one- and two-dendrimer mixed clusters. A surfactant molecule is considered absorbed if it is part of an absorbed micelle (see below). For the *G*3 molecules and ε* = 1.25, only minor surfactant absorption is observed, as indicated by the sharp maximum of the histogram near *s* = 0 and its rapid decline to zero. The surfactants exhibit a tendency to bind to the dendrimers at ε* = 1.5 and ε* = 1.6, as reflected by the broad histogram displaying multiple local maxima and minima. The oscillatory nature of the histogram for small dendrimers may result from a combination of structural irregularities, microscale electrostatic interactions, and the limited number of available absorption sites. For higher dendrimer generations (*G*5 and *G*7), the histogram is bell-shaped and dominated by a single peak, reflecting pronounced surfactant absorption. The shift of the histogram toward larger *s* values with increasing ε* indicates strengthening of this effect. This observation is consistent with the behavior of the mean fraction of absorbed surfactants, *f*_*s*_ = ⟨s⟩/*n*_*s*_, plotted in Figure 2b as a function of ε*. Here, ⟨*s*⟩ denotes the mean number of absorbed surfactant chains, calculated using *H*(*s*). It can be observed that for a given *G*, *f*_*s*_ increases from small values as ε* rises, with higher-generation dendrimers exhibiting a faster growth rate. Conversely, an increase in *f*_*s*_ is also noted with increasing *G* at a given ε*, with the trend becoming more pronounced at higher ε* values (see the inset of Figure 2b). In particular, we find that for the largest *G*7 dendrimers and ε* = 1.6, nearly 80% of the surfactant chains are absorbed. Thus, our simulations demonstrate that dendrimer–surfactant complexation is enhanced for larger dendrimers, which can be attributed to their higher charge. As we show below, the ε*- driven increase in *f*_*s*_ correlates with the observation that micelles in mixed clusters are mostly multichain structures.

For our further analysis, we distinguish between three types of aggregates. The first type consists of free (unbound) micelles formed solely by surfactant molecules. A micelle is defined as a set of surfactant chains, in which each chain is bound to at least one other chain within the set. Two surfactant molecules are considered bound if a pair of tail monomers from the two distinct chains are separated by less than r* = 1.5. A special case of micelles is single surfactant chains, referred to as unimers. The second type of aggregate consists of mixed clusters. We define a mixed cluster as a set of mutually bound micelles and dendrimers that cannot be divided into two disjoint, nonempty subsets. A micelle and a dendrimer are considered bound if the head of at least one surfactant chain from the micelle is within r* = 1.5 of at least one terminal group of the dendrimer. In general, in a mixed cluster containing more than one dendrimer, some micelles are bound to multiple dendrimers. These micelles act as bridges connecting distinct dendrimers, while others are bound to individual dendrimers within the cluster. We refer to the former as bridge micelles and the latter as corona micelles. Specifically, in mixed clusters containing only one dendrimer, all micelles are corona micelles. The third type of aggregate consists of a single dendrimer with no surfactants bound.

**Figure 3:**
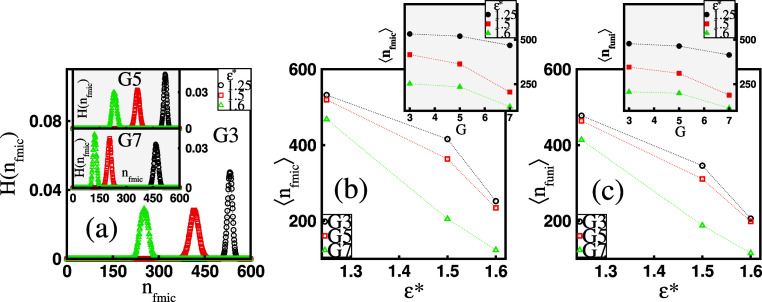
(a) Histogram, *H*(*n*_fmic_), of the number of free micelles, *n*_fmic_, for varying surfactant hydrophobicity, ε*, and fixed dendrimer generation, *G*. The mean number of (b) free micelles, ⟨*n*_fmic_⟩, and (c) free unimers, ⟨*n*_funi_⟩, as functions of ε* for fixed *G*. The insets in (b) and (c) show ⟨*n*_fmic_⟩ and ⟨*n*_funi_⟩ as functions of *G* at a fixed ε*, respectively.

**Figure 4:**
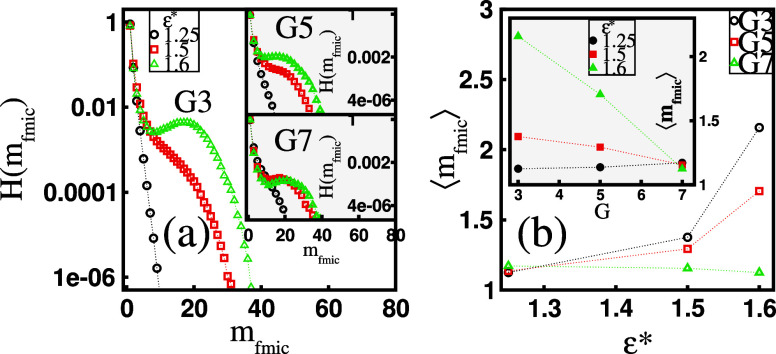
(a) Histogram, *H*(*m*_fmic_), of the mass, *m*_fmic_, of free micelles at varying surfactant hydrophobicity, ε*, and fixed dendrimer generation, *G*, presented on a log-lin scale. (b) The mean mass of free micelles, ⟨*m*_fmic_⟩, as a function of ϵ* for a given *G*. The inset in (b) shows ⟨*m*_fmic_⟩ as a function of *G* at a fixed ϵ*.

Note that, as shown in Figure 2, not all surfactant chains are bound to the dendrimers, and some remain in solution as free micelles. To inspect these micelles, in Figure 3a, we display the histogram, *H*(*n*_fmic_), of the number of free micelles, *n*_fmic_, for varying ε* at a fixed *G*. The histograms are bell-shaped, and the position of their maximum moves toward a smaller number of free micelles as ε* increases. As a result, the mean number of free micelles, ⟨*n*_fmic_⟩, in the solution decreases for more hydrophobic surfactants, as shown in Figure 3b. In line with the histogram’s trend in response to changes in *G*, we also observe a reduction in ⟨*n*_fmic_⟩ as this parameter increases at a fixed ε* (see the inset in Figure 3b). As demonstrated in Figure 3c, similar trends in response to variations in ε* and *G* are also found for the mean number of free unimers, ⟨*n*_funi_⟩. It is worth noting that ⟨*n*_fmic_⟩ does not significantly exceed ⟨*n*_funi_⟩ for a given ε* value and *G*. Specifically, our data show that the ratio ⟨*n*_funi_⟩/⟨*n*_fmic_⟩ falls within the ranges of 0.8 and 0.9, indicating that most free micelles are unimers. The latter finding is also confirmed in Figure 4a, which presents the histogram, *H*(*m*_fmic_), of the mass, *m*_fmic_, of free micelles (expressed as the number of unimers) at varying ε* values. It can be seen that at ε* = 1.25, only small free micelles are present in the solution, with unimers predominating. For more hydrophobic surfactants, the population of unimers still dominates. However, there is a finite probability of free massive micelles forming, as indicated by the broadening of the histogram. As a result, the mean mass of free micelles, ⟨*m*_fmic_⟩, does not significantly exceed 1 (see Figure 4b). In particular, for the *G*7 molecules, we find that ⟨*m*_fmic_⟩ ≈ 1 at each ε*. For the *G*3 and *G*5 dendrimers, free micelles, on average, become larger with increasing ε*, reaching an average value of ⟨*m*_fmic_⟩ ≈ 2 at ε* = 1.6. Note that the increase in ⟨*m*_fmic_⟩ is most pronounced for smaller dendrimers, which correlates with the ε* dependence of *f*_*s*_ (see Figure 2b). For low-generation dendrimers, the extent of surfactant absorption is weaker compared to that in larger dendrimers. This results in a higher concentration of free surfactants in the solution, which, in turn, enhances their assembly. As demonstrated in the inset of Figure 4b, this correspondence is also observed when considering ⟨*m*_fmic_⟩ as a function of *G* at a fixed ε*. In this case, ⟨*m*_fmic_⟩ decreases with *G* as larger dendrimers absorb more surfactants, especially at higher ε* values (see the inset in Figure 2b). Therefore, the concentration of free surfactants decreases, and their tendency to self-assemble diminishes.

**Figure 5:**
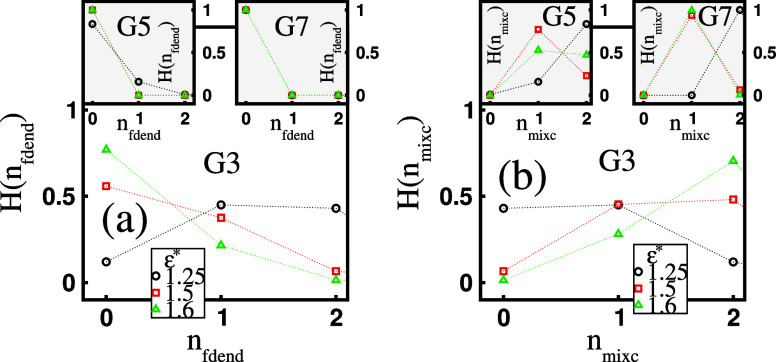
(a) Histogram, *H*(*n*_fdend_), of the number of free (unbound) dendrimers, *n*_fdend_, and (b) histogram, *H*(*n*_mixc_), of the number of mixed clusters, *n*_mixc_, for varying surfactant hydrophobicity, ε*, at a fixed dendrimer generation, *G*.

Up to this point, we have shown that both free micelle formation and surfactant absorption by the dendrimers take place. However, we have yet to investigate the potential mixed cluster types that could result from the latter effect. To explore this, it is helpful to first examine the histogram *H*(*n*_fdend_), which represents the number of free (unbound) dendrimers, *n*_fdend_, in the system. In Figure 5a, we display *H*(*n*_fdend_) for varying ε* values and fixed *G*. Note that, for our system of two dendrimers, the situation *n*_fdend_ = 0 describes states with either one mixed cluster containing both dendrimers or two separate mixed clusters, each holding one dendrimer. The scenario *n*_fdend_ = 1 represents conditions with one free dendrimer and one mixed cluster comprising the other dendrimer. Finally, the case of *n*_fdend_ = 2 indicates instances with two free dendrimers. For the G3 molecules and ε* = 1.25, the probability of states occurring in which the surfactant is absorbed by both dendrimers is close to zero. Only conformations in which the surfactant is absorbed by one of the two dendrimers or by neither occur with nearly equal probabilities, approximately 0.5. For ε* = 1.5 and ε* = 1.6, the scenario changes. In this case, conformations with two free dendrimers are very unlikely, whereas conformations with surfactant molecules bound to one or both dendrimers occur with finite probabilities. For the *G*5 and *G*7 dendrimers, predominantly two mixed clusters, each composed of one dendrimer, or a single mixed cluster consisting of two dendrimers, form with a high probability even at ε* = 1.25. States with one or two free dendrimers are very unlikely to occur (see the insets in Figure 5a). To complement our presentation of possible clusters, we also display in Figure 5b the histogram *H*(*n*_mixc_), representing the number of mixed clusters, *n*_mixc_. For the *G*3 dendrimers at ε* = 1.25, states completely devoid of mixed clusters and those with a single mixed cluster occur with nearly equal probability of approximately 0.5, whereas states with two mixed clusters are unlikely. At ε* = 1.5 and ε* = 1.6, states with one or two mixed clusters dominate, while those without mixed clusters are nearly absent. For the *G*5 and *G*7 molecules, the absence of mixed clusters is not observed, even at ε* = 1.25 (see the insets in Figure 5b). Under these ε*-conditions, the most likely outcome is the formation of two mixed clusters. At ε* = 1.5, the *G*5 dendrimers and surfactants form one and two mixed clusters, with the former being the most probable. At ε* = 1.6, both outcomes are equally likely. For the *G*7 dendrimers, states with a single mixed cluster dominate at both ε* = 1.5 and ε* = 1.6.

**Figure 6:**
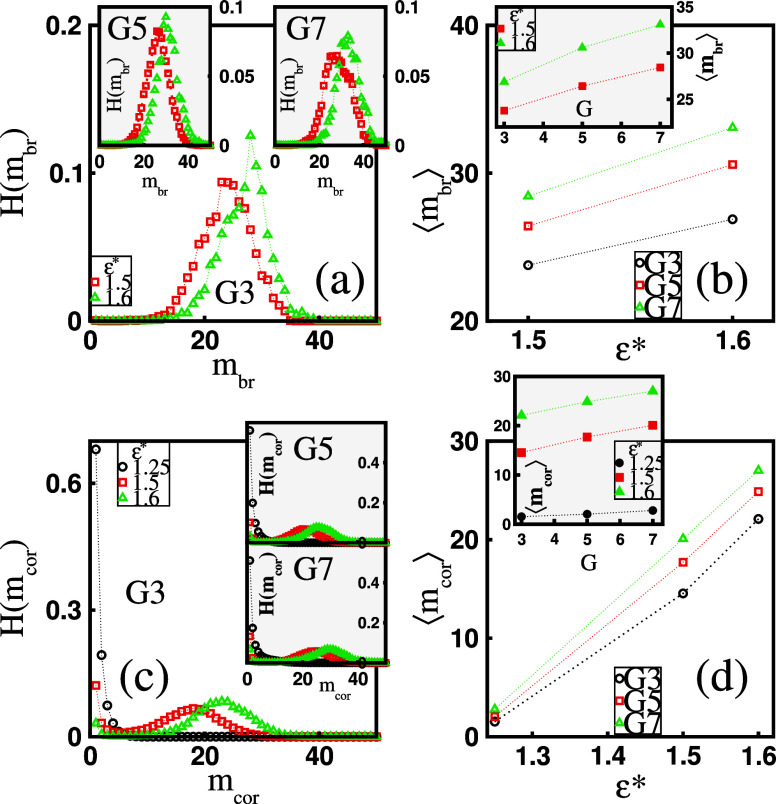
(a) Histogram, *H*(*m*_br_), of the bridge micelle mass, *m*_br_, and (c) histogram, *H*(*m*_cor_), of the corona micelle mass, *m*_cor_, for varying surfactant hydrophobicity, ε*, at a fixed dendrimer generation, *G*. (b) The mean bridge micelle mass, ⟨*m*_br_⟩, and (d) the mean corona micelle mass, ⟨*m*_cor_⟩, as functions of ε* at a fixed *G*. The insets in panels (b) and (d) display ⟨*m*_br_⟩ and ⟨*m*_cor_⟩, respectively, as functions of *G* at a fixed ε*.

Next, we examine the size properties of bridge and corona micelles within mixed clusters without distinguishing between different mixed cluster types. Figure 6a shows the histogram *H*(*m*_br_) of the bridge micelle mass, *m*_br_, for a fixed *G* and varying ε*. Results for ε* = 1.25 are omitted due to insufficient statistical reliability in the simulation data (see below). It can be observed that *H*(*m*_br_) exhibits a bell-shaped distribution with a distinct maximum. As ε* increases, the distribution shifts to the right, indicating a tendency of the surfactant to form larger bridge micelles. This effect is further illustrated in Figure 6b, which depicts the mean mass of bridge micelles, ⟨*m*_br_⟩, as a function of ε* for a fixed *G*. Notably, unlike free micelles, bridge micelles are, on average, significantly more massive. For the examined dendrimers and surfactant hydrophobicities, ⟨*m*_br_⟩ falls within the range 23 ≲ ⟨*m*_br_⟩ ≲ 33. The inset in Figure 6b shows ⟨*m*_br_⟩ as a function of *G*. It can be observed that with a fixed ε*, ⟨*m*_br_⟩ tends to increase for larger dendrimers. The histogram *H*(*m*_cor_) of the corona micelle mass, *m*_cor_, for a fixed *G* and varying ε*, is shown in Figure 6c. For weakly hydrophobic chains at ε* = 1.25, most corona micelles exist as unimers, as indicated by the histogram’s peak at *m*_cor_ ≈ 1 and its sharp decline at larger *m*_cor_ values. At ε* = 1.5 and ε* = 1.6, the dominance of unimeric corona micelles diminishes and larger corona micelles begin to form. This is indicated by the histogram becoming bell-shaped with a local maximum at *m*_cor_ ≫ 1 and the disappearance of the peak at *m*_cor_ ≈ 1. Thus, like bridge micelles, corona micelles tend to become more massive at larger ε*, as also shown by the shift in the location of the histogram’s maximum. Such a shift is consistent with the ε*-driven increase in the mean mass of corona micelles, ⟨*m*_cor_⟩, presented in Figure 6d. Specifically, our data reveal that ⟨*m*_cor_⟩ changes from ⟨*m*_cor_⟩ ≈ 1 at ε* = 1.25 to ⟨*m*_cor_⟩ ≈ 25 at ε* = 1.6. The effect of *G* on ⟨*m*_cor_⟩ at a fixed ε* value is shown in the inset of Figure 6d. It confirms that, on average, light corona micelles dominate for weakly hydrophobic surfactants. In contrast, for more hydrophobic chains, corona micelles are already large when assembled with low-generation dendrimers, and a significant increase in ⟨*m*_cor_⟩ is observed with increasing *G*.

**Figure 7:**
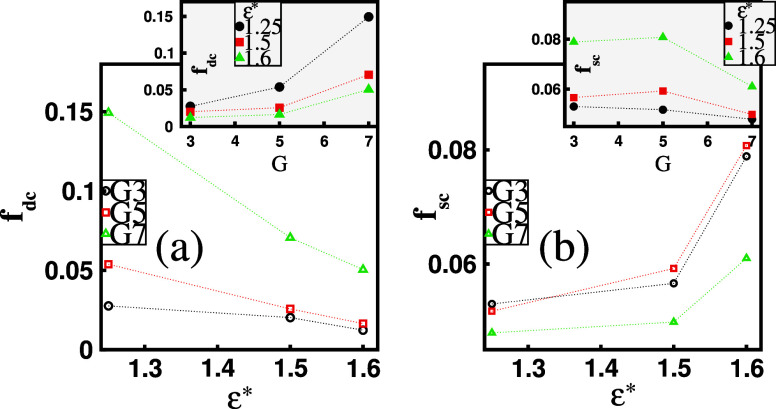
Mean total fraction of (a) dendrimer counterions, *f*_dc_, and (b) surfactant counterions, *f*_sc_, condensed on free dendrimers, free micelles, and mixed clusters as a function of surfactant hydrophobicity, ε*, for a given dendrimer generation, *G*. The insets display *f*_dc_ and *f*_sc_ as functions of *G* for a fixed ε*.

With the formation of complexes, the next factor to consider is the behavior of counterions. In Figure 7, we display the mean total fraction of dendrimer counterions, *f*_dc_ = ⟨*n*_dc_⟩/(*n*_*d*_*N*_t_), and surfactant counterions, *f*_sc_ = ⟨*n*_sc_⟩/*n*_*s*_, condensed on free dendrimers, free micelles, and mixed clusters as a function of ε* for fixed *G*. Here, ⟨*n*_dc_⟩ and ⟨*n*_sc_⟩ represent the mean total numbers of condensed dendrimer and surfactant counterions, respectively. A counterion is considered condensed if its distance from at least one bead in an aggregate is less than r* = 1.5. Figure 7a shows that increasing ε* leads to a reduction in *f*_dc_, primarily due to the formation of mixed clusters containing large surfactant micelles. These micelles function as multivalent anions, replacing dendrimer counterions in the process of condensing onto the clusters. Moreover, as the micelles in the mixed clusters grow larger, their attraction to surfactant counterions strengthens, further promoting the condensation of surfactant counterions (Figure 7b). As shown in the inset of Figure 7a, *f*_dc_ increases with *G*, primarily because larger dendrimers, with their greater charge, attract more dendrimer counterions. For the same reason, the opposite effect is observed for surfactant counterions (see the inset in Figure 7b). However, if we take into account the absolute values of both *f*_dc_ and *f*_sc_, we find that only small fractions of counterions are condensed. Thus, we find that a vast majority of counterions remain unbound and diffuse into the solution as free ions.

In summary, our analysis reveals that the system under consideration comprises various aggregates, including free surfactant micelles, dendrimers, and mixed clusters. Free micelles predominantly form small assemblies, even with strongly hydrophobic surfactants, due to the substantial absorption of surfactants by dendrimers. This absorption significantly reduces the concentration of free surfactants, limiting their self-assembly. In contrast, both bridge and corona micelles within mixed clusters increase in size as the surfactant hydrophobicity rises. Large micelles consist of a dense core of hydrophobic surfactant tails surrounded by a corona, where the charged surfactant heads are concentrated.^16,17^ These micelles act as multivalent anions, replacing dendrimer counterions through a strong Coulomb attraction to the terminal monomers. Our findings suggest that mixed cluster formation is driven by a surfactant-induced ion exchange process.^24,25^ In this process, counterions gain translational entropy, while multivalent ions bind to the dendrimer’s charges, lowering the system’s energy. This ion exchange mechanism highlights the strong influence of the surfactant hydrophobicity on its assembly with dendrimers.

In the remaining part of this article, we classify aggregates from a different perspective and provide a more detailed description of their statistical properties. Our focus shifts to analyzing the probabilities of various aggregates forming. We calculate the average number and mass of bridge and corona micelles within these aggregates and their effective charge. In the following analysis, aggregates consisting of *s* surfactants and *d* dendrimers are denoted by pairs (*s*,*d*), where 0 ≤ *s* ≤ *n*_*s*_ and 0 ≤ *d* ≤ *n*_*d*_. For *s* = 0, only the case of *d* = 1 is considered. Pairs (*s*,0) refer to free micelles consisting of *s* > 0 surfactant chains, while a pair (0,1) denotes free, single dendrimers. Pairs with *s*, *d* > 0 refer to mixed clusters, where, as mentioned above, surfactants can form corona and bridge micelles. Furthermore, it is convenient to index the aggregates with a single integer. Specifically, for *s* > 0, the pair (*s*,*d*) is uniquely represented by *n*_*s*,*d*_ = *dn*_*s*_ + *s*, while the pair (0,1) is represented by *n*_0,1_ = 0. Note that, given *n*_*s*,*d*_ > 0, *s* and *d* can be determined using the following formulas:  and *s* = *n*_*s*,*d*_ – *dn*_*s*_ (where *a* div *b* denotes integer division of *a* by *b*). In the subsequent analysis, cluster index *n*_*s*,*d*_ is utilized as the variable for histogram representation. This approach allows us to identify the types of clusters present in the system and determine their occurrence probabilities with *n*_*s*,*d*_ serving as the grouping criterion for the histogram.

**Figure 8:**
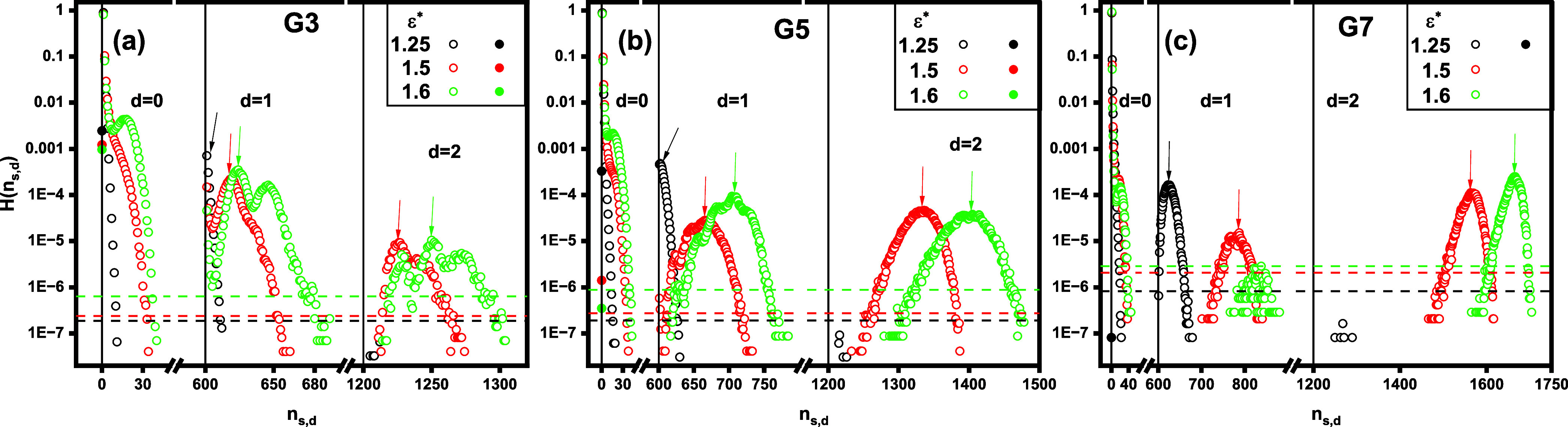
Histogram, *H*(*n*_*s*,*d*_), of the number of aggregates containing *s* surfactant chains and *d* dendrimers, plotted as a function of the aggregate index, *n*_*s*,*d*_. The data is presented on a log–linear scale for varying surfactant hydrophobicity ε*, with the dendrimer generation *G* held constant. The black solid vertical separators indicate the intervals on the *n*_*s*,*d*_-axis corresponding to aggregates with *d* = 0, *d* = 1, and *d* = 2 dendrimers, respectively. The dashed horizontal separators on the *H*(*n*_*s*,*d*_)-axis indicate the probabilities corresponding to ten occurrences of aggregates at a given ε*. The arrows indicate the locations of the highest local maxima in *H*(*n*_*s*,*d*_) for *d* = 1 and *d* = 2, which correspond to the favored mixed clusters. The open symbols represent mixed clusters (*n*_*s*,*d*_ = *dn*_*s*_ + *s* > 0, 0 < *s* ≤ *n*_*s*_, 0 < *d* ≤ *n*_*d*_) and free micelles (*n*_*s*,*d*_ = *s* > 0, 0 < *s* ≤ *n*_*s*_, *d* = 0), while the full symbols represent free dendrimers (*n*_*s*,*d*_ = 0).

**Figure 9:**
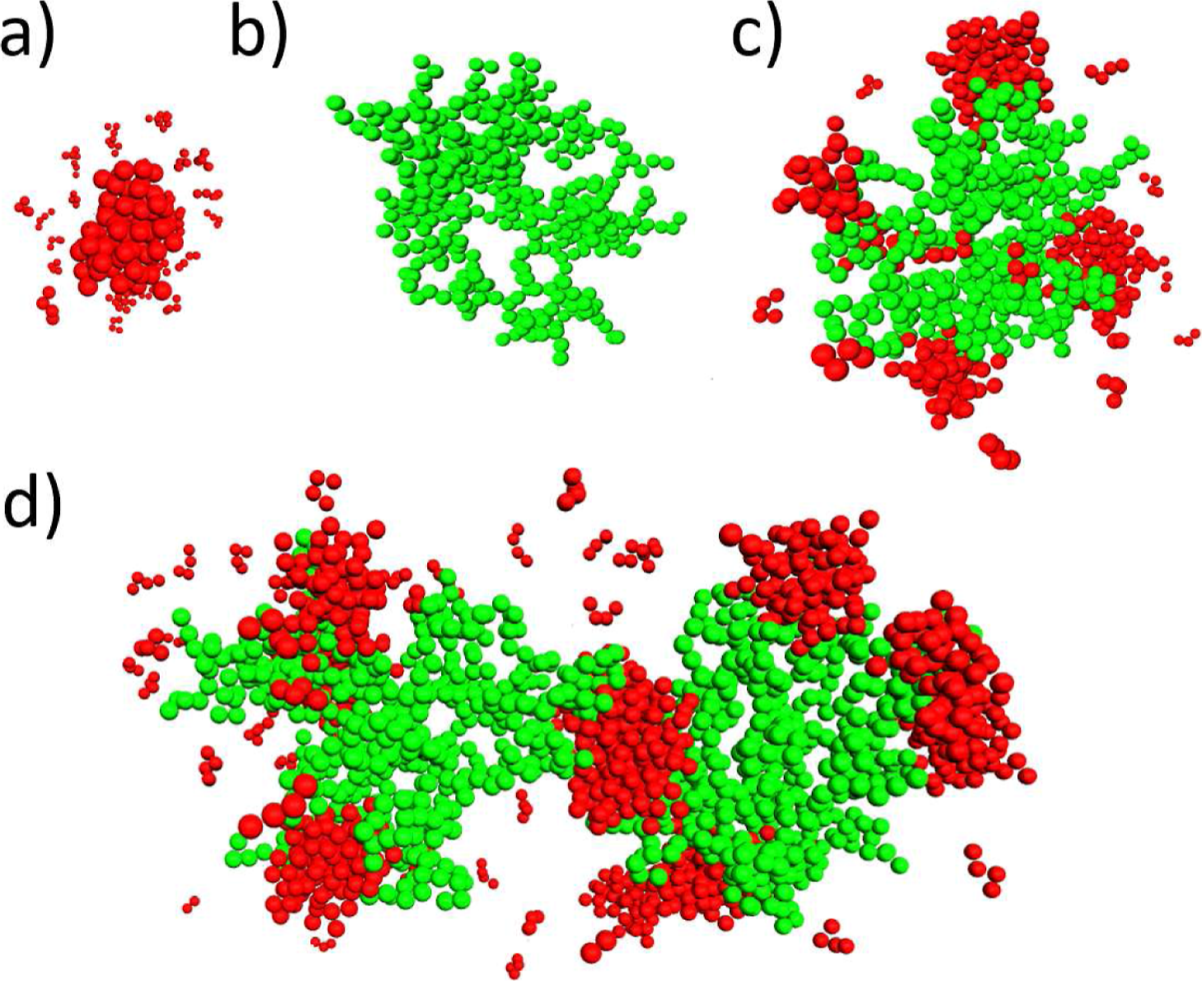
Molecular dynamics snapshots of various dendrimer–surfactant aggregate types: (a) free surfactant micelles without dendrimers (*d* = 0), (b) single dendrimers (*n*_0,1_ = 0), (c) mixed clusters consisting of single dendrimers (*d* = 1), and (d) mixed clusters consisting of two dendrimers (*d* = 2). The dendrimers and surfactant chains are displayed in green and red, respectively. For better visibility, dendrimer and surfactant counterions are not shown.

In Figure 8, we show the histogram *H*(*n*_*s*,*d*_), representing the number of aggregates containing *s* surfactant chains and *d* dendrimers as a function of the aggregate index *n*_*s*,*d*_. The data are displayed for varying ε* values with a fixed dendrimer generation *G*. The plots are divided into three ranges (by the solid vertical separators) numbered by *d*, which denotes the number of dendrimers in the aggregates. The *d* = 0 range corresponds to unbound, free micelles (cf. Figure 9a), where *n*_*s*,0_ = *s* > 0 also represents their mass. The case *n*_0,1_ = 0 refers to free dendrimers (cf. Figure 9b). The *d* = 1 and *d* = 2 ranges correspond to mixed clusters consisting of one and two dendrimers, respectively, along with *s* > 0 surfactant chains (cf. Figure 9c,d). The histogram primarily reveals the occurrence of various supramolecular structures, including free micelles, dendrimers, and mixed clusters of both types, each with finite probabilities. This finding aligns qualitatively with experimental studies,^1,26^ where a diverse array of structures was observed, thereby reinforcing the validity of the proposed model. In particular, free unimers (*n*_1,0_ = 1) occur with the highest probability of approximately 0.9. This observation aligns with our aforementioned finding that the vast majority of aggregates present in the system are unbound unimers. The solution may also contain more massive free micelles. However, the probability of their occurrence decreases sharply with increasing mass *n*_*s*,0_.

**Figure 10:**
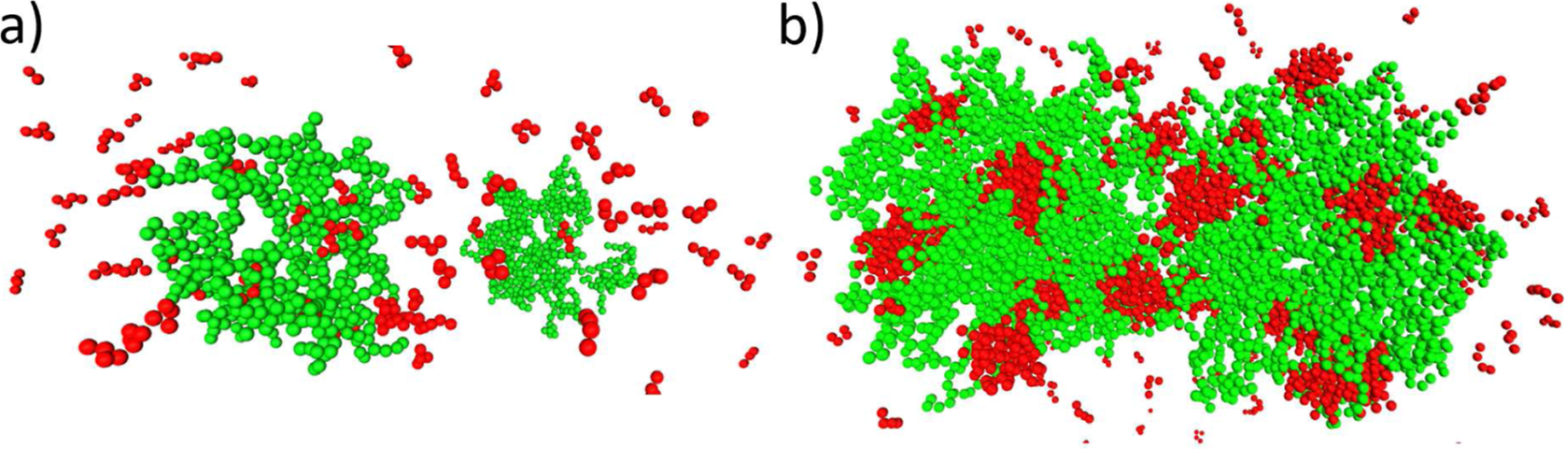
Molecular dynamics snapshots of (a) *G*5 dendrimers at ε* = 1.25 and (b) *G*7 dendrimers at ε* = 1.6. The dendrimers and surfactant chains are displayed in green and red, respectively. For better visibility, dendrimer and surfactant counterions are not shown.

With respect to the influence of ε* on *H*(*n*_*s*,*d*_), Figure 8 shows that more hydrophobic surfactants are more likely to self-assemble into larger free micelles. On the other hand, within the ranges of mixed clusters (the case of *d* = 1 and *d* = 2 in Figure 8), the histograms are either bell-shaped or half-bell-shaped, featuring one or more local maxima. This trend indicates that for a given *d* > 0, mixed clusters contain surfactant chains within specific numerical ranges, while clusters outside these limits are unlikely to occur. Given that these ranges are relatively wide, the amount of complexed surfactant molecules can vary significantly. It is important to note that due to the presence of maxima in the histogram, certain numbers of surfactant chains in the aggregates, *s*_f_, are statistically favored. For example, at ε* = 1.5, one *G*5 dendrimer forms mixed clusters with surfactants for 601 ≲ *n*_*s*,1_ ≲ 730 (*d* = 1, 1 ≲ *s* ≲ 130), with a maximum at  (*d* = 1, *s*_f_ ≈ 66). In the following, we refer to these aggregates as favored aggregates or favored mixed clusters. For more hydrophobic chains, the histogram shifts to the right, indicating that a greater number of surfactants tend to associate with the dendrimers with a specific probability. In particular, the favored mixed clusters contain more surfactant molecules as ε* increases. Interestingly, the histogram also shows that at ε* = 1.6, single *G*7 dendrimer mixed clusters occur with an extremely low probability, while aggregates containing two dendrimers are predominantly observed. As a result, in the regime of strongly hydrophobic chains and high-generation dendrimers, the solution is predominantly composed of free unimers and two-dendrimer mixed clusters (see the snapshot displayed in Figure 10b).

**Figure 11:**
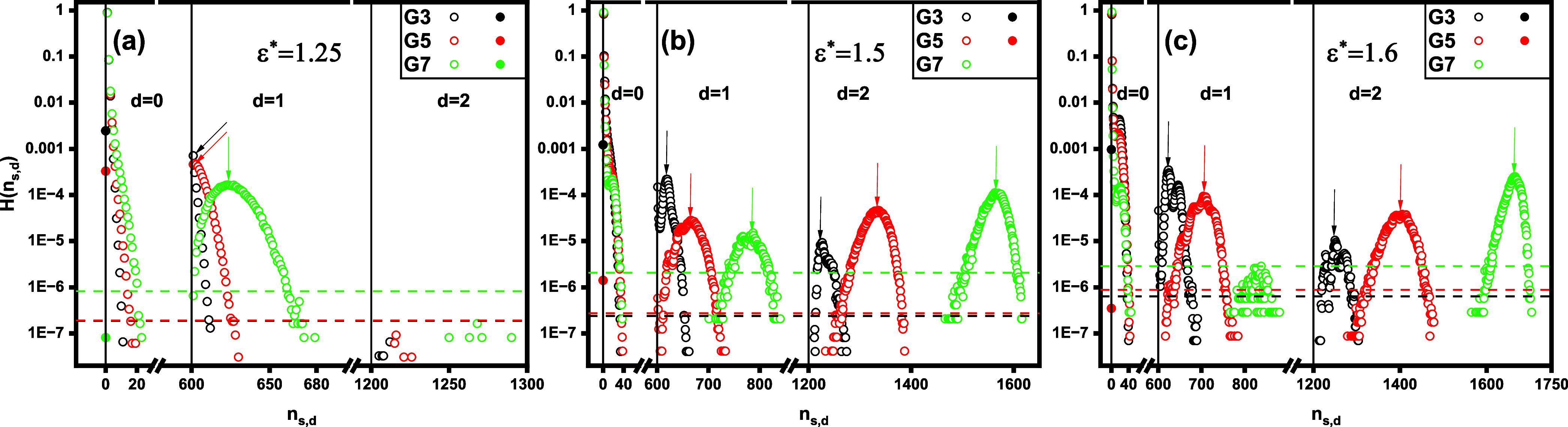
Histogram, *H*(*n*_*s*,*d*_), of the number of aggregates containing *s* surfactant chains and *d* dendrimers, plotted as a function of the aggregate index, *n*_*s*,*d*_. The data is presented on a log–linear scale for varying dendrimer generations, *G*, with a fixed surfactant hydrophobicity, ε*. The black solid vertical separators indicate the intervals on the *n*_*s*,*d*_-axis corresponding to aggregates with *d* = 0, *d* = 1, and *d* = 2 dendrimers, respectively. The dashed horizontal separators on the *H*(*n*_*s*,*d*_)-axis indicate the probabilities corresponding to ten occurrences of aggregates at a given G. The arrows indicate the locations of the highest local maxima in *H*(*n*_*s*,*d*_) for *d* = 1 and *d* = 2, which correspond to the favored mixed clusters. The open symbols represent mixed clusters (*n*_*s*,*d*_ = *dn*_*s*_ + *s*, 0 < *s* ≤ *n*_*s*_, 0 < *d* ≤ *n*_*d*_) and free micelles (*n*_*s*,*d*_ = *s*, 0 < *s* ≤ *n*_*s*_, *d* = 0), while the filled symbols represent free dendrimers (*n*_*s*,*d*_ = 0).

**Figure 12:**
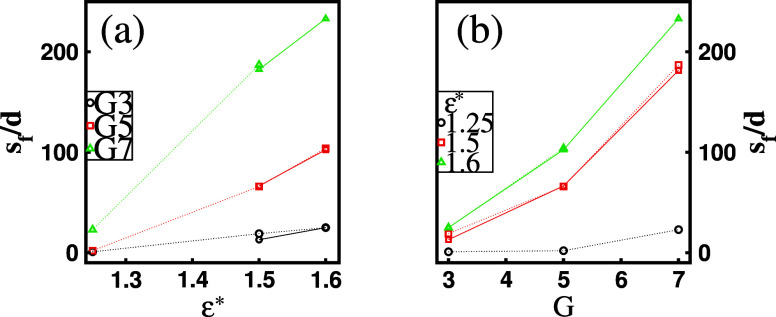
Number of surfactants per dendrimer, *s*_*f*_/*d*, in a favored mixed cluster as a function of (a) surfactant hydrophobicity ε* for a fixed dendrimer generation *G* and (b) *G* for a fixed ε*. The dotted lines correspond to *d* = 1, while the solid lines correspond to *d* = 2.

In Figure 11, we display the histogram *H*(*n*_*s*,*d*_) for fixed ε* and varying *G*. We observe that for fixed *d*, an increase in *G* shifts the histogram toward larger *n*_*s*,*d*_ values. We attribute this effect to a stronger Coulomb attraction between the surfactant and the higher charge of the larger-generation dendritic polyelectrolytes. Note that the histogram demonstrates that, at ε* = 1.25, the probability of forming mixed clusters with two dendrimers is significantly lower compared to that of complexes containing only one dendrimer (see Figure 11a). In this scenario, two-dendrimer clusters are very unlikely to occur in the solution. Consequently, we find that, in the limit of weakly hydrophobic chains, mixed clusters containing one dendrimer are statistically favored (see snapshot in Figure 10a). To further support our analysis of the impact of ε* and *G* on the dendrimer–surfactant assembly, Figure 12 illustrates the number of surfactants per dendrimer, *s*_f_/*d*, in the favored mixed clusters as functions of these parameters. The results reveal a slight increase for low-generation dendrimers and weakly hydrophobic chains, while a more pronounced rise is observed for high-generation dendrimers and strongly hydrophobic chains.

Note that, in general, for a mixed cluster of the (*s*, *d*) type, the set of *s* surfactant chains is divided into corona micelles and bridge micelles. Both the number and mass of these micelles are characterized by specific distributions, with *s* ≈ ⟨*n*_br_⟩(*n*_*s*,*d*_)⟨*m*_br_⟩(*n*_*s*,*d*_) + ⟨*n*_cor_⟩(*n*_*s*,*d*_)⟨*m*_cor_⟩(*n*_*s*,*d*_). Here, ⟨*n*_br_⟩(*n*_*s*,*d*_) and ⟨*m*_br_⟩(*n*_*s*,*d*_) denote the mean number and the mean mass of bridge micelles, respectively, while ⟨*n*_cor_⟩(*n*_*s*,*d*_) and ⟨*m*_cor_⟩(*n*_*s*,*d*_) represent the mean number and the mean mass of corona micelles as functions of aggregate index *n*_*s*,*d*_. The averaging is performed over various realizations of (*s*, *d*)-type clusters that appear more than ten times during the simulations, excluding those with fewer occurrences due to insufficient statistical reliability. This procedure is also applied to the mean values of other quantities discussed below.

**Figure 13:**
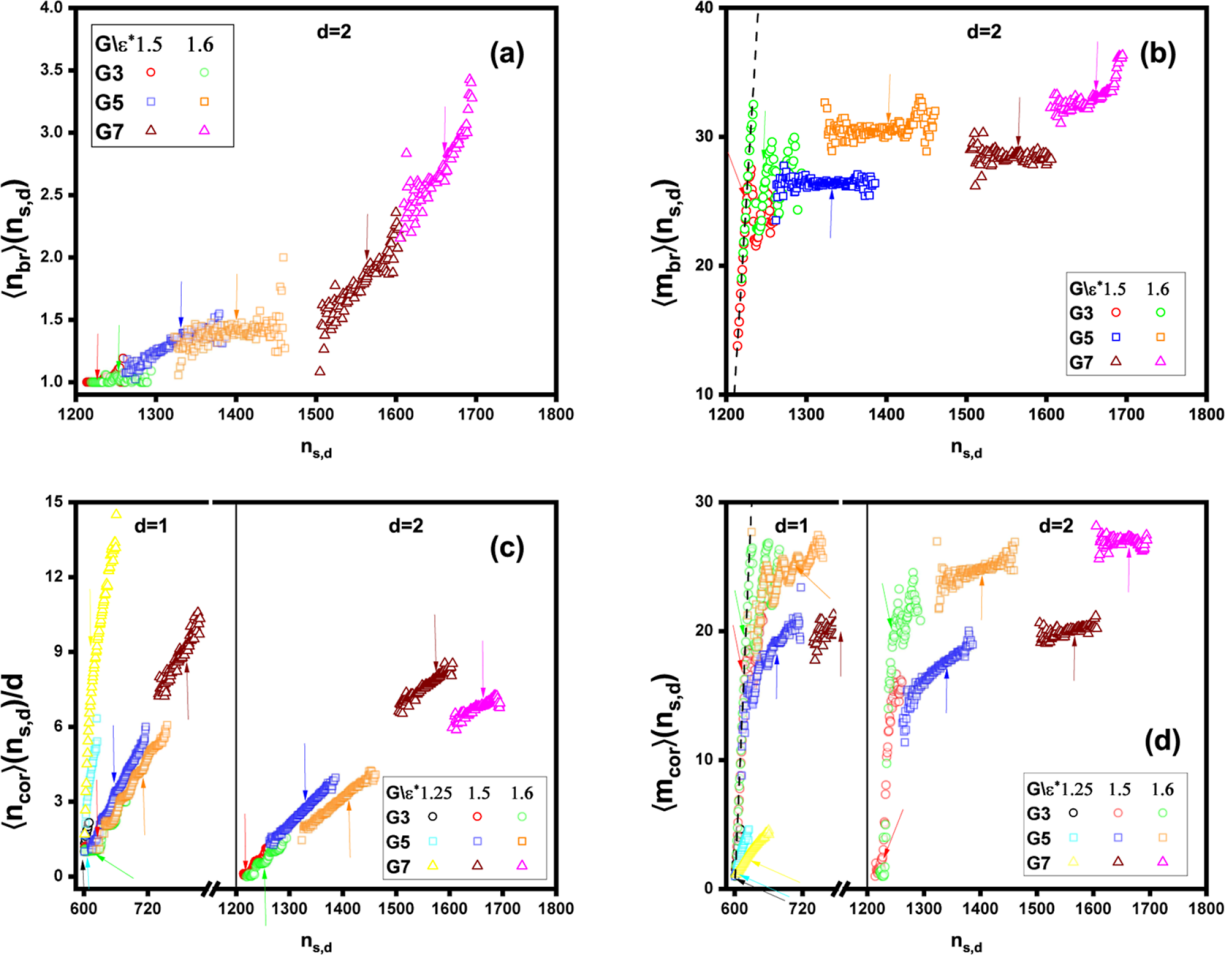
(a) The mean number of bridge micelles ⟨*n*_br_⟩(*n*_*s*,*d*_), (b) the mean mass of bridge micelles ⟨*m*_br_⟩(*n*_*s*,*d*_), (c) the mean number of corona micelles per dendrimer in a mixed cluster ⟨*n*_cor_⟩(*n*_*s*,*d*_)/*d*, and (d) the mean mass of corona micelles ⟨*m*_cor_⟩(*n*_*s*,*d*_) as functions of the aggregate index *n*_*s*,*d*_, for varying surfactant hydrophobicity ε* and dendrimer generation *G*. In panels (b) and (d), the dashed line indicates the condition ⟨*m*_br_⟩(*n*_*s*,*d*_) = *s* and ⟨*m*_cor_⟩(*n*_*s*,*d*_) = *s*, respectively. The arrows indicate the mean values for the favored mixed clusters (see Figures 8 and 11). In panels (c) and (d), the black solid vertical separators indicate the intervals on the *n*_*s*,*d*_-axis corresponding to aggregates with *d* = 1 and *d* = 2 dendrimers, respectively.

**Figure 14:**
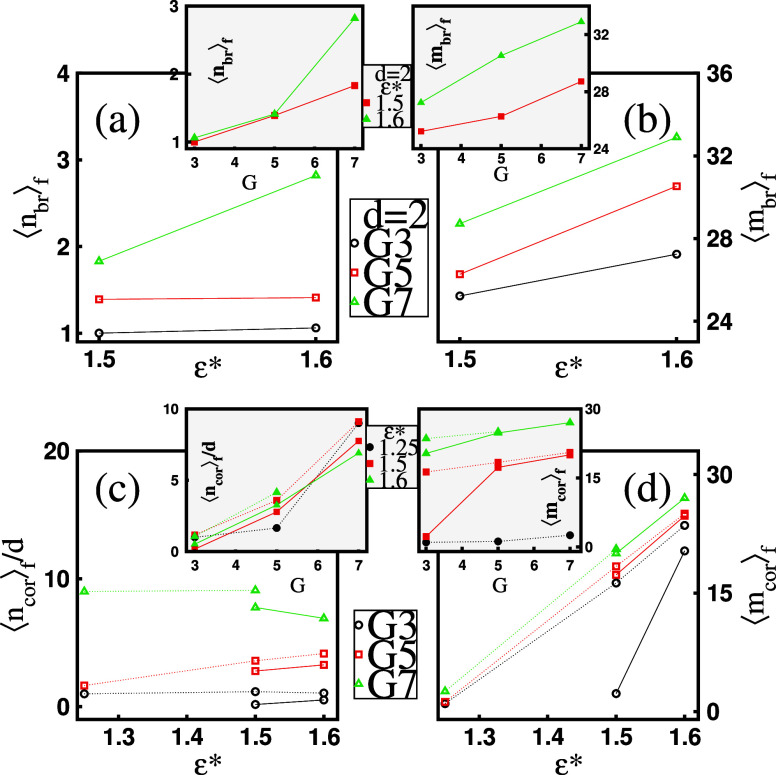
Mean values of (a) the number of bridge micelles, , in a favored mixed cluster with two dendrimers, (b) the mass of bridge micelles, , in a favored mixed cluster with two dendrimers, (c) the number of corona micelles per dendrimer in a favored mixed cluster, , and (d) the mass of corona micelles, , in a favored mixed cluster as functions of surfactant hydrophobicity ε* at a given dendrimer generation *G*. The insets present the same quantities as functions of *G* at a fixed ε*. The dotted lines correspond to *d* = 1, while the solid lines correspond to *d* = 2.

In Figure 13a, we display the mean number of bridge micelles, ⟨*n*_br_⟩(*n*_*s*,*d*_), as a function of the cluster index *n*_*s*,*d*_. According to our above discussion, only conformations recorded at ε* = 1.5 and 1.6 provide satisfactory statistics (see Figure 11a). It can be seen that for given *G* and ε*, ⟨*n*_br_⟩(*n*_*s*,*d*_) increases gradually as the number of clustered surfactants increases. Thus, on average, two-dendrimer mixed clusters with a greater number of surfactants tend to contain more bridge micelles, a trend that is especially pronounced for *G*7 molecules. It is important to note that, as previously mentioned, mixed clusters with a specific index dominate the population. These clusters provide the highest statistical reliability and most accurately represent the properties of the aggregates, assuming that the cluster index, *n*_*s*,*d*_, is used as the criterion for histogram grouping. To illustrate this, Figure 14a depicts the mean number of bridge micelles, , within the favored two-dendrimer mixed clusters. For *G*3 and *G*5 dendrimers,  remains nearly constant with ε*, ranging between . In contrast, for the largest *G*7 dendrimer,  increases with ε*, rising from  to . On the other hand,  increases from  to  when transitioning from *G*3 to *G*7 at a fixed ε* (see the inset in Figure 14a). Consequently, the average number of bridge micelles in the favored mixed clusters remains relatively small, typically on the order of a few. The mean mass of bridge micelles, ⟨*m*_br_⟩(*n*_*s*,*d*_), as a function of *n*_*s*,*d*_ is displayed in Figure 13b. Across all values of *G* and ε*, these micelles are found to be substantial in size, with 13 ≲⟨*m*_br_⟩(*n*_*s*,*d*_) ≲ 35. For the *G*5 and *G*7 dendrimers at a fixed ε*, ⟨*m*_br_⟩(*n*_*s*,*d*_) remains largely unaffected by the amount of absorbed surfactant, aside from minor fluctuations. However, the smallest *G*3 dendrimer exhibits a distinct profile for ⟨*m*_br_⟩(*n*_*s*,*d*_), characterized by an approximately piecewise linear trend. This trend alternates between increasing and decreasing segments, creating a zigzag pattern. Notably, near *n*_*s*,*d*_ = 1200, ⟨*m*_br_⟩(*n*_*s*,*d*_) follows the relation ⟨*m*_br_⟩(*n*_*s*,*d*_) ≈ *n*_*s*,*d*_ – *dn*_*s*_ = *s*. This behavior suggests that all surfactants in the clusters containing two *G*3 dendrimers self-assemble into a single bridge micelle of mass *s*, eliminating the presence of corona micelles. The mean mass of bridge micelles, , in the statistically favored mixed clusters is presented in Figure 14b. The figure reveals that, in this case, surfactant chains predominantly form massive bridge micelles with , and this tendency becomes more pronounced as ε* increases. A similar pattern is observed for a fixed ε* as the dendrimer generation, *G*, increases (see the inset in Figure 14b).

Regarding corona micelles, Figure 13c illustrates their mean number per dendrimer, ⟨*n*_cor_⟩(*n*_*s*,*d*_)/*d*, as a function of the aggregate index, *n*_*s*,*d*_. For fixed values of *d*, ε*, and *G*, this quantity generally increases with the amount of surfactant in mixed clusters, although some local fluctuations are evident. Figure 14c displays the mean number of corona micelles per dendrimer, , as a function of ε* for the favored mixed clusters. The impact of ε* is generally weak, except for corona micelles bound to single *G*5 dendrimers (*d* = 1), where a noticeable increase from  to  is observed. In contrast, as shown in the inset of Figure 14c, higher-generation dendrimers tend to have a greater average number of corona micelles at a fixed ε*. Specifically,  increases from approximately 1 to 8 as the dendrimer generation rises from *G*3 to *G*7. Figure 13d presents the mean mass of corona micelles, ⟨*m*_cor_⟩(*n*_*s*,*d*_), for varying ε* and *G*. For mixed clusters containing two *G*5 or *G*7 dendrimers (*d* = 2), the corona micelles are predominantly massive at ε* = 1.5 and ε* = 1.6, with ⟨*m*_cor_⟩(*n*_*s*,*d*_) showing a slight increase with *n*_*s*,*d*_, except for *G*5 at ε* = 1.5, where the increase is more pronounced. In clusters with two *G*3 dendrimers, a slight rise in surfactant content near *n*_*s*,*d*_ = 1200 leads to a sharp transition in ⟨*m*_cor_⟩(*n*_*s*,*d*_), increasing from approximately 1 to 20, indicating the occurrence of light and massive corona micelles. For single-dendrimer clusters (*d* = 1), massive corona micelles also form at ε* = 1.5 and ε* = 1.6. For *G*5, ⟨*m*_cor_⟩(*n*_*s*,*d*_) oscillates sharply with *n*_*s*,*d*_, whereas for *G*7, it fluctuates around 20. Notably, for *G*3 dendrimers, corona micelle assembly follows ⟨*m*_cor_⟩(*n*_*s*,*d*_) ≈ *n*_*s*,*d*_ – *dn*_*s*_ = *s* near *n*_*s*,*d*_ = 600, forming mixed clusters with a single *G*3 dendrimer and one corona micelle. In contrast, for weakly hydrophobic surfactants (ε* = 1.25), only small corona micelles are absorbed by the dendrimers, with ⟨*m*_cor_⟩(*n*_*s*,*d*_) increasing modestly with surfactant content, ranging from approximately 1 to 5. To further elaborate on corona micelles, Figure 14d presents their mean mass, , as a function of ε* for aggregates with the most probable composition. For the favored one-dendrimer aggregates (*d* = 1), a transition from light to massive corona micelles occurs as surfactant hydrophobicity increases, with  rising from approximately 1 to 25 across all dendrimer generations. A similar trend is evident for the favored mixed clusters containing two dendrimers (*d* = 2), where  at ε* = 1.5 reaches approximately 20 for *G*5 and *G*7 but remains around 1 for *G*3. The inset in Figure 14d highlights  as a function of *G* with ε* fixed. For weakly hydrophobic surfactants in one-dendrimer aggregates, corona micelles remain light. In contrast, for strongly hydrophobic surfactants, corona micelles are consistently massive as *G* increases. Generally,  exhibits only a weak dependence on *G*, except at *d* = 2 and ε* = 1.5, where a sharp increase is observed, with  rising from nearly 1 to 20 as dendrimer generation increases from *G*3 to *G*5.

**Figure 15:**
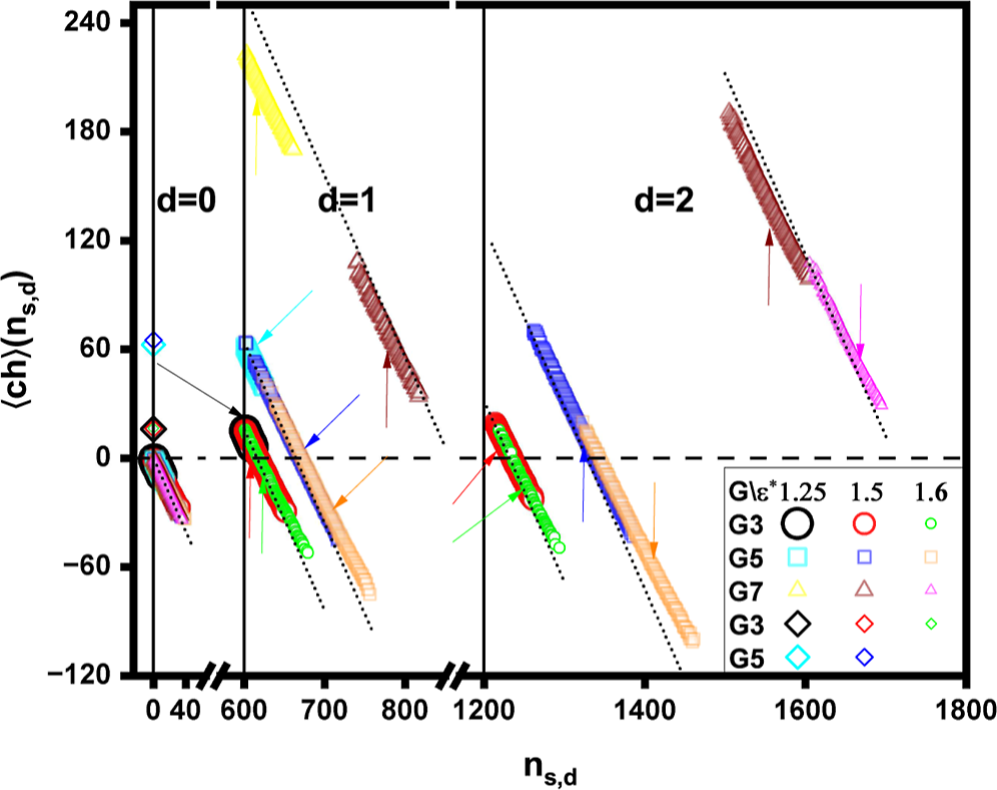
Mean effective charge of aggregates, ⟨*ch*⟩(*n*_*s*,*d*_), versus the cluster index, *n*_*s*,*d*_, for varying surfactant hydrophobicity, ε*, and dendrimer generation, *G*. The dashed horizontal line indicates ⟨*ch*⟩(*n*_*s*,*d*_) = 0. The black solid vertical separators indicate the intervals on the *n*_*s*,*d*_-axis corresponding to aggregates with *d* = 0, *d* = 1, and *d* = 2 dendrimers, respectively.The dotted lines indicate the condition . The arrows indicate the mean effective charge of the favored aggregates (see Figures 8 and 11). The spheres, squares, and triangles represent mixed clusters (*n*_*s*,*d*_ = *dn*_*s*_ + *s*, 0 < *s* ≤ *n*_*s*_, 0 < *d* ≤ *n*_*d*_) and free micelles (*n*_*s*,*d*_ = *s*, 0 < *s* ≤ *n*_*s*_, *d* = 0), while the diamonds represent free dendrimers (*n*_*s*,*d*_ = 0).

In Figure 15, we present the mean effective charge of aggregates, ⟨*ch*⟩(*n*_*s*,*d*_), given by ⟨*ch*⟩(*n*_*s*,*d*_) = *dN*_*t*_ – *n*_*s*,*d*_ + *dn*_*s*_ + ⟨*n*_*sc*_⟩(*n*_*s*,*d*_) – ⟨*n*_*dc*_⟩(*n*_*s*,*d*_), as a function of the cluster index *n*_*s*,*d*_. The figure also includes the cases of free micelles (*d* = 0, *s* > 0) and dendrimers (*n*_0,1_ = 0). Here, ⟨*n*_*sc*_⟩(*n*_*s*,*d*_) and ⟨*n*_dc_⟩(*n*_*s*,*d*_) denote the mean numbers of condensed surfactant and dendrimer counterions, respectively, for a cluster with index *n*_*s*,*d*_. For a given ε*, *G*, and *d*, ⟨*ch*⟩(*n*_*s*,*d*_) exhibits a linear decrease with *n*_*s*,*d*_. This behavior primarily stems from the previously discussed minimal condensation of the counterions. Specifically, the data reveal that ⟨*ch*⟩(*n*_*s*,*d*_) closely approximates its value without condensed counterions, given by ⟨*ch*⟩(*n*_*s*,*d*_) = *dN*_*t*_ – *n*_*s*,*d*_ + *dn*_*s*_. Due to the high positive charge of the *G*7 dendrimer, the mean effective charge of clusters containing these molecules remains positive, irrespective of whether the surfactants are weakly or strongly hydrophobic. In contrast, for the *G*3 and *G*5 dendrimers, within the ε* range where massive bridge and corona micelles are present (ε* = 1.5 and ε* = 1.6), the number of surfactant chains associated with the dendrimers surpasses their overall charge. As a result, ⟨*ch*⟩(*n*_*s*,*d*_) becomes negative, and the overall charge of the micelles within the mixed clusters dominates. Additionally, free dendrimers retain their intrinsic positive charge, *N*_t_, while free micelles carry a negative charge, −*s*.

**Figure 16:**
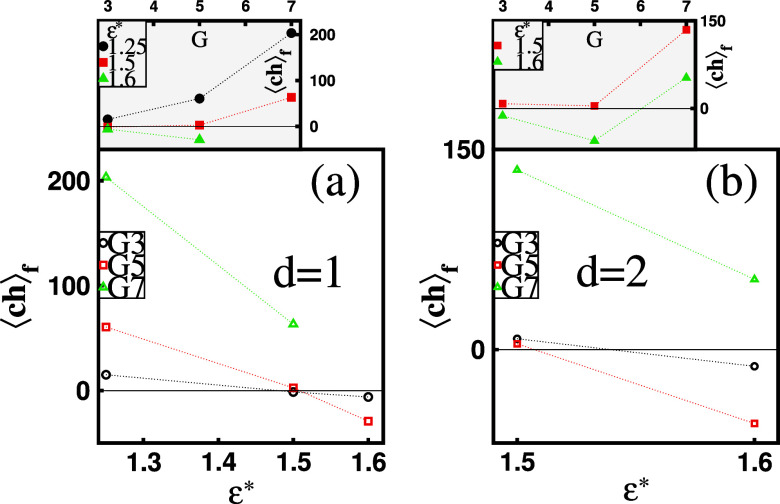
Mean effective charge of favored mixed clusters, ⟨*ch*⟩_*f*_, for (a) *d* = 1 and (b) *d* = 2 dendrimers within them, as functions of surfactant hydrophobicity, ε*, for a given dendrimer generation, *G*. The insets in panels (a) and (b) depict ⟨*ch*⟩_*f*_ as a function of *G* at a fixed ε*. The solid horizontal lines represent ⟨*ch*⟩_*f*_ = 0.

Finally, in Figure 16, we present the mean effective charge of favored mixed clusters, ⟨*ch*⟩_*f*_, as a function of ε* for a given dendrimer generation *G*. The figure highlights the phenomenon of overcharging in mixed clusters containing *G*3 and *G*5 dendrimers, which becomes more pronounced with increasing ε* for both single- and two-dendrimer configurations. This overcharging effect is absent for *G*7 molecules due to their inherently large positive charge. The insets in Figure 16 further illustrate ⟨*ch*⟩_*f*_ as a function of *G* at a fixed ε*. These plots show a general increase in ⟨*ch*⟩_*f*_, from approximately ⟨*ch*⟩_*f*_ ≈ 0 for *G*3 dendrimers to ⟨*ch*⟩_*f*_ ≫ 0 for *G*7 molecules. Notably, for ε* = 1.25 (*d* = 1) and ε* = 1.5 (*d* = 1, 2), the trend is monotonic, with ⟨*ch*⟩_*f*_ being dominated by the dendrimer charge. For ε* = 1.6 and *d* = 2, it becomes nonmonotonic, with ⟨*ch*⟩_*f*_ values reaching a negative minimum for *G*5 and turning positive again for G7. This observation indicates the occurrence of charge inversion in these clusters. In the regime of small dendrimers and strongly hydrophobic surfactants, ⟨*ch*⟩_*f*_ is primarily determined by contributions from bound surfactants. Conversely, for high-generation dendrimers, this trend is reversed, and the intrinsic positive charge of the dendrimers becomes the dominant factor in the overall effective charge of the favored clusters.

## 4 Summary

In this study, we investigated the complexation behavior of anionic surfactants, terminally charged cationic dendrimers, and counterions using Langevin dynamics simulations. Our findings reveal that the system consists of free surfactant micelles, free dendrimers, and mixed dendrimer–surfactant clusters. The mixed clusters are composed of either two dendrimers with bridge and corona micelles or one dendrimer with corona micelles only. Structural analysis indicates that free surfactant micelles predominantly exist as unimers, while those in mixed clusters grow larger with an increasing surfactant hydrophobicity. Additionally, most counterions remain uncondensed and diffuse in solution, resulting in translational entropy. Based on this, the formation of mixed clusters can be understood as a manifestation of the ion exchange mechanism, where large micelles act as multivalent ions, replacing dendrimer counterions in their binding to dendrimers. This process lowers the system’s energy and, together with counterion release, drives dendrimer–surfactant assembly.

To analyze the system at a microscopic level, we introduced aggregate indexing, specifying the number of dendrimers and surfactants within the aggregates. Histograms based on this index reveal that the population of mixed clusters is influenced by surfactant hydrophobicity and dendrimer generation: one-dendrimer aggregates dominate for weakly hydrophobic surfactants, whereas two-dendrimer aggregates become predominant in the limit of strongly hydrophobic surfactants and high-generation dendrimers. The histograms also indicate statistically favored aggregates with the most probable composition. These aggregates, characterized by the mean number and mass of bridge and corona micelles, show that these micelles typically form in small numbers per dendrimer and become more massive with increasing surfactant hydrophobicity.

Regarding charge properties, the mean effective charge of the aggregates is largely determined by the combined charges of dendrimers and surfactants, with minor contributions from counterion condensation. For high-generation dendrimers, this charge remains positive across the index range, whereas lower-generation dendrimers exhibit an inversion point, where the charge shifts from positive to negative as the number of complexed surfactants increases. Charge inversion is also observed in the favored aggregates containing low-generation dendrimers as surfactant hydrophobicity increases as well as in the favored aggregates with dendrimers of increasing generations in the case of strongly hydrophobic surfactants.

Our simulations were limited to two cationic dendrimers immersed in a solution of anionic surfactants at a fixed concentration. Studying two dendrimers represents a natural progression from our previous work on single dendrimer–surfactant systems. This configuration, while simple, is nontrivial and provides a fundamental starting point for exploring the formation of multidendrimer–surfactant complexes. Future research could extend to several directions. For instance, at significantly higher surfactant concentrations, free micelles may no longer be predominantly unimers, and unabsorbed micelles of increasing mass are likely to form. Investigating such conditions would allow for a broader exploration of the charge inversion effect in mixed clusters, particularly in high-generation dendrimers. Additionally, incorporating more than two dendrimers into the system could give rise to a more diverse array of mixed clusters. Thus, examination of the effects of varying dendrimer and surfactant concentrations presents both an intriguing and challenging avenue for further study.
